# Tebentafusp: T Cell Redirection for the Treatment of Metastatic Uveal Melanoma

**DOI:** 10.3390/cancers11070971

**Published:** 2019-07-11

**Authors:** Bertil E. Damato, Joseph Dukes, Howard Goodall, Richard D. Carvajal

**Affiliations:** 1 Nuffield Department of Clinical Neurosciences, University of Oxford, John Radcliffe Hospital, Oxford OX3 9DU, UK; 2 Immunocore Limited, 101 Park Drive, Milton Park, Abingdon, Oxon OX14 4RY, UK; 3 Department of Medicine, Columbia University Irving Medical Center, Herbert Irving Comprehensive Cancer Center, Herbert Irving Pavilion, 161 Fort Washington Avenue, HIP 9, New York, NY 10032, USA

**Keywords:** metastatic uveal melanoma, immunotherapy, tebentafusp, T cell, clinical data, preclinical data, ImmTAC platform, T cell receptor, anti-CD3 bispecific

## Abstract

Metastatic disease from uveal melanoma occurs in almost 50% of patients suffering from this ocular tumour, with median survival from development of symptoms being around 1 year. In contrast to cutaneous melanoma, kinase inhibitors and immune checkpoint inhibitors are usually ineffective in patients with metastatic uveal melanoma. Tebentafusp is a novel form of immunotherapy based on the immune-mobilising monoclonal T cell receptor against cancer (ImmTAC) platform, which comprises a soluble T cell receptor that is fused to an anti-CD3 single-chain variable fragment. The T cell receptor domain of tebentafusp targets cells present a human leukocyte antigen-A*02:01 complexed with a peptide derived from the melanoma-associated antigen gp100, which is expressed strongly by melanoma cells, weakly by normal melanocytes and minimally by other tissues. The anti-CD3 domain recruits CD3+ T cells (and, indirectly, other immune cells), redirecting these to the melanoma cells. The most common adverse events with tebentafusp are manageable and usually transient. Early survival data in patients with metastatic uveal melanoma are promising when considered alongside historical data. Based on these encouraging results, a randomised study comparing tebentafusp to investigator’s choice of therapy in metastatic uveal melanoma is ongoing.

## 1. Introduction

Almost 50% of patients with uveal melanoma (UM) develop metastatic disease (i.e., metastatic uveal melanoma (mUM)), despite successful treatment of the primary ocular tumour [1]. Metastatic disease usually involves the liver and is commonly fatal within a year of the onset of symptoms [2]. Systemic therapy for metastases rarely prolongs life [3]; however, recent clinical trials have shown encouraging results with tebentafusp (formerly IMCgp100), a first-in-class, bispecific, fusion protein that redirects CD3+ T cells to gp100-expressing melanoma cells, inducing cytolysis [4,5].

In this review, we describe UM, cancer immunotherapy and the research that has translated tebentafusp from the laboratory to clinical trials.

## 2. UM Biology

UM threatens patients with visual handicap, a painful eye, facial disfigurement and untimely death from a metastatic disease [6,7,8]. The incidence is approximately six cases per million people per year [9]. Approximately 90% of UMs involve the choroid; the remainder being confined to the iris or the ciliary body [6,8,10]. Presentation almost always occurs in adulthood, peaking at around the age of 60 years [8,10]. Males are more commonly affected than females [8,9,10]. The large majority of patients are white, and a light-coloured iris is a risk factor [8]. Other predisposing factors include ocular melanocytoma, congenital ocular melanocytosis and the *BAP1* tumour predisposition syndrome [8].

Approximately 80% of UMs arise from mutations in the G alpha pathway (via mutually exclusive mutations in *GNAQ*, *GNA11*, *PLCB4* or *CYSLTR2*) [11]. This is in contrast to cutaneous melanoma (CM), the most common form of melanoma [12], which commonly harbours *BRAF* and *NRAS* mutations [8,13]. In further contrast to CM, exposure to ultraviolet light is not considered a risk factor for choroidal and ciliary body melanomas [8]. Familial inheritance is also rare with UM, with less than 5% of patients having an inherited component [14].

Treatment of primary UM is aimed at preventing the metastatic spread and, if possible, conserving the eye and useful vision [6,15]. Eye-conserving therapeutic modalities include various forms of radiotherapy, laser therapy and surgical resection [6]. Radiotherapy is administered either with a radioactive plaque, proton beam, or a stereotactic technique, all of which deliver high doses of radiation at the tumour, while minimising the collateral damage to the optic nerve, fovea, lens, and other ocular structures [8,16].

Surgical resection can be performed either in a piecemeal fashion with a vitreous cutter passed through the retina (i.e., endoresection) or by en-bloc excision through a large scleral window (i.e., exoresection and eyewall resection) [17]. Laser therapy (consisting of transpupillary thermotherapy or photodynamic therapy) has a higher rate of local tumour recurrence than other modalities and is, therefore, administered only as an adjunct therapy, except for very small tumours where it is used as the primary treatment [6]. Up to a third of patients require enucleation, either as primary treatment (because the tumour is advanced) or after attempted ocular conservation because of local tumour recurrence or other complications, such as painful neovascular glaucoma after radiotherapy (i.e., toxic tumour syndrome) [18,19]. Quality of life after treatment for UM is usually good—even after enucleation—with any psychological problems usually caused by unrelated factors [20,21]. The Collaborative Ocular Melanoma Study reported no survival differences between patients treated with radiotherapy and those treated with enucleation [22]; however, it is not yet known whether metastasis occurs because of tumour recurrence after eye-conserving therapy [15,23].

## 3. Metastatic Disease

Despite successful ablation or removal of the ocular tumour, almost 50% of patients with UM develop metastatic disease [1]. It is not known whether ocular treatment ever influences survival and, if so, in whom [15]. Metastatic spread occurs haematogenously and almost always involves the liver, with lung, bone and other organs less frequently affected [2,24]. Metastatic disease develops almost exclusively in patients who have tumours that show chromosome 3 deletion, *BAP1* loss or a class 2 gene expression profile (as defined by expression of 12 genes that influence the metastatic spread) [25,26,27]. The time required for developing overt metastatic disease is shorter in patients with a larger tumour, especially if it shows a higher grade of malignancy (e.g., with epithelioid cytomorphology and a high mitotic count) [28,29].

The median survival time after detection of metastases from UM is around 1 year, which varies according to the disease-free interval from primary tumour treatment, size of the largest metastasis, the performance status and liver function tests [2]. There is a general paucity of effective therapies; the National Comprehensive Cancer Network guidelines for UM recommending entry into a clinical trial as the preferred option for metastatic disease [30]. As shown in Table 1, a number of therapeutic approaches have been studied in mUM and very little success has been observed to date, except with liver resections [31].

As a result of limited treatment options, outcomes for patients with metastatic disease have not improved significantly for over 30 years, and more effective therapies are urgently needed [35].

## 4. Immunotherapy and UM

The first indication that stimulating the immune system can help fight cancer came in 1893, when Coley discovered that injecting a patient with dead bacteria could cause a fever that induced a tumour regression [43].

We now recognise immune evasion to be one of the hallmarks of cancer [44,45]. Cancerous mutations can generate non-self neoantigens (non-native proteins), so that the cancer cells might be recognised and killed by cytotoxic T cells [46]; however, T cell immunity to cancer neoantigens is frequently limited. This is because cancer neoantigens are often closely related to self-antigens and T cells that are reactive against self-antigens are deleted by thymic selection. This selection process, known as central tolerance, prevents self-reactive T cells from generating autoimmune diseases [47]. Some auto-reactive T cells escape the thymus and these are regulated by a number of peripheral tolerance mechanisms. For example, cytotoxic T cells are prevented from attacking normal cells by expressing deactivating (anergy-inducing) molecules, such as programmed cell death protein-1 (PD-1) and cytotoxic T-lymphocyte antigen 4 (CTLA-4). In addition, any T cells that recognise self-antigens are normally inhibited, inactivated, or deleted by ‘tolerogenic’ dendritic cells, which play a vital role in maintaining central and peripheral tolerance, either directly or via the generation and activation of regulatory T cells (known as Treg cells) [48]. Cytotoxic T cells are also then suppressed by Treg cells [49].

A cancer that is clinically detectable has, by definition, evaded immune-mediated destruction. T cell eradication of cancer cells displaying high numbers of neoantigens creates a selection pressure for cells displaying fewer neoantigens (i.e., ‘immunoediting’) [50]. Furthermore, malignant tumours can create an immunosuppressive environment, for example, by expressing programmed death-ligand 1 (PD-L1) to downregulate the activity of cytotoxic T cells, as well as attracting Treg cells and secreting immunosuppressive cytokines [51].

Immunotherapy aims to circumvent immune evasion by tumour cells and to induce immune-mediated tumour cell death. One of the earliest forms of immunotherapy deployed interleukin (IL)-2 [52], a T cell growth factor that stimulates expansion of cytotoxic T cells and natural killer cells [53]. IL-2 is an effective therapy for metastatic melanoma and metastatic renal cell carcinoma, which has been approved for use in both cancers [52]. More recently, immune checkpoint inhibitors have been developed, which block PD-L1 (e.g., durvalumab, atezolizumab), PD-1 (e.g., pembrolizumab, nivolumab), or CTLA-4 (e.g., ipilimumab), thereby preventing the suppression of cytotoxic T cells [54]. These have proved highly effective in the treatment of CM and other cancers [55]. The success of these inhibitors in a number of tumour types has led to a search for novel checkpoints—T cell immunoreceptor with Ig and ITIM domains (TIGIT) [56], T cell immunoglobulin and mucin domain-3 (TIM-3) [57] and lymphocyte-activation gene 3 (LAG-3) [58], which appear to be promising therapeutic targets.

Other innovative approaches include a variety of adoptive T cell therapies, such as those using tumour-infiltrating lymphocytes (TIL) [59], engineered T cell receptors (TCRs) [60] and chimeric antigen receptors (CAR) on T cells [61]. While CAR T cell therapies have been approved for use in haematological malignancies [62,63], and TIL therapy has shown promise in mUM [59], these forms of therapy are expensive and require a highly skilled team to prepare the tumour-specific cytotoxic T cells over several weeks [64,65,66]. Furthermore, enhancement of adoptive T cell therapy by immunodepletion and high-dose IL-2 therapy can cause severe complications [67]. Serious morbidity can also be caused by immune reactions against healthy tissues, the release of cytokines [68], and (in the case of CAR T cell therapy) anaphylaxis against murine antigens [69]. Immune receptor agonists are another novel class of immunotherapies that promote signalling leading to immune-activation. These include CD122 agonists, which bind CD122 (the IL-2 receptor) and induce CD8+ T cell and natural killer cell expansion [70]; OX-40 agonists, which provide costimulatory signals to CD4+ and CD8+ T cells [71]; and glucocorticoid-induced tumour necrosis factor receptor family related gene (GITR) agonists, which reduce Treg-mediated immune suppression and activate CD8+ T cells [72].

UMs have a number of features that make them less amenable to current immunotherapies. Algazi et al. performed a meta-analysis focusing on patients with mUM receiving immune checkpoint inhibitors against PD-1 and PD-L1, and reported an objective response rate (ORR) of only 3.6% and a median progression-free survival (PFS) as short as 2.6 months [41]. These poor results were confirmed by a meta-analysis by Khoja et al., which showed a median PFS of 2.8 months in patients receiving immunotherapies [42] Among the potential biological explanations for this limited efficacy is the finding that UM has one of the lowest tumour mutational burden amongst all cancers, therefore, the neoantigen display is limited [14,41,73,74]. This might explain why checkpoint inhibitors, when tested in patients with mUM, have shown little clinical benefit [41]. This is in contrast to CMs, which have a very high tumour mutational burden [73] and which show excellent responses to checkpoint inhibitors [75]. Additionally, PD-L1 expression has been shown to positively correlate with responses to anti-PD-(L)1 inhibitors [73,76]. Compared to metastatic CM, mUM tissue samples show lower PD-L1 expression [77], which might further account for the limited efficacy of checkpoint inhibitors in mUM. PD-L1 expression appears to be higher in primary UM than in liver lesions [78], which could explain recent results showing promising efficacy for pembrolizumab in patients without bulky liver lesions [74]. However, as discussed previously, liver lesions are almost ubiquitous in mUM; therefore these data are compatible with the observed low response rate of checkpoint inhibitors in mUM. Overall, the low PD-L1 expression and low mutational burden in UM might explain the apparent lack of activity of checkpoint inhibitors in this tumour. Beyond checkpoint inhibition, Chandran et al. reported promising results in 2017 with autologous TIL and high-dose interleukin-2 in patients with mUM in a single-centre Phase II study; seven of 20 evaluable patients demonstrated objective tumour regression (35%; 95% CI: 16–59%), but this trial has since been terminated. [59] Van Loenen et al. have developed a T-cell receptor-modified T cells targeting preferentially expressed antigen in melanoma (PRAME) [60] and Forsberg et al. have developed CAR-T cells that eradicate uveal melanoma cells in animal models [61]; however, to our knowledge no clinical results have yet been reported.

## 5. Development of ImmTAC Molecules

**Figure 1:**
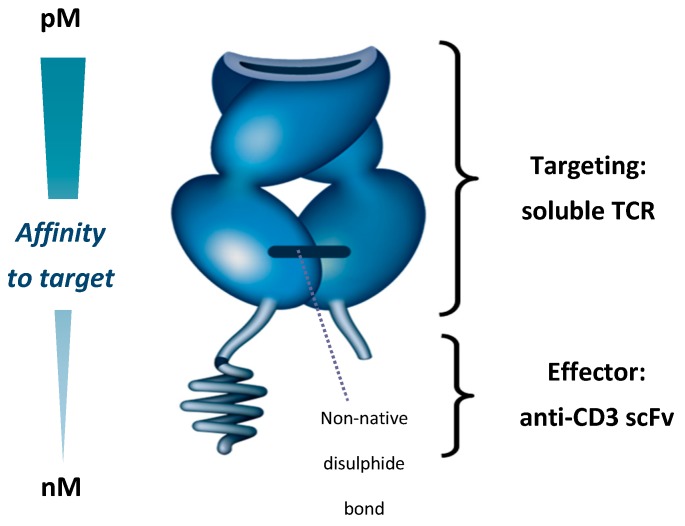
Immune-mobilising monoclonal T cell receptor against cancer (ImmTAC) molecule showing T cell receptor (TCR) targeting domain and effector anti-CD3 scFv. scFv—Single-chain variable fragment; TCR, T cell receptor.

The ImmTAC (Immune-mobilising monoclonal TCRs against cancer) platform is a first-in-class immunotherapy platform with a novel mechanism of action (MoA). ImmTAC molecules bind cells that present a peptide derived from an antigen of interest, and recruit T cells to lyse the target cells [79]. To achieve this, ImmTAC molecules are composed of a TCR targeting domain and a single-chain variable fragment (scFv) anti-CD3 effector domain. The TCR targeting domain binds peptides presented on the cell surface as a human leukocyte antigen (HLA)-peptide (pHLA) complex, while the anti-CD3 effector domain engages and activates CD3+ T cells (Figure 1) [79]. Peptide antigens presented by HLA represent approximately 90% of the proteome, providing ImmTAC molecules with a large range of potential targets [80]. The anti-CD3 domain allows ImmTAC molecules to engage T cells that are not specific for cancer cells. This redirection of T cells should enable ImmTAC molecules to overcome the shortcomings of immune checkpoint inhibitors in mUM, as checkpoint inhibitors require pre-existing cancer-specific T cells in order to induce cancer cell lysis [79].

Native TCRs, which are membrane-bound, have an affinity for specific pHLA complexes typically in the range of 10–300 µM [81]. To create a therapeutically efficacious ImmTAC molecule, three challenges had to be addressed—(a) producing a soluble TCR that was stable; (b) enhancing ImmTAC molecule affinity to tumour antigens; and (c) triggering an antitumour immune response. 

The native TCR is a heterodimer consisting of two transmembrane α and β chains linked by a membrane–proximal disulphide bond [82,83]. While it is possible to produce a soluble TCR by removing the transmembrane elements of the two chains, this process breaks the disulphide bond so that the resulting heterodimer easily disassociates and is unable to bind its target [83]. To overcome this issue, an artificial disulphide bond was created between the two chains by mutating two proximal residues to cysteine. This results in a soluble, stable TCR that forms the basis of ImmTAC molecule-targeting [79].

Secondly, natural TCRs have a low affinity for their target antigens, with K_d_ values typically in the range of 10–300 µM [81]. Increasing this affinity is essential for creating therapeutically useful TCRs, given that the target tumour cells can display as few as 10 pHLA complexes per cell [84]. Additionally, soluble TCRs lack the avidity provided by co-receptor engagement in a normal T cell–tumour cell interaction, meaning higher affinity is needed for the soluble TCR to engage its target [79]. By mutating the hypervariable regions of the TCR, affinity to target is increased 10^6^-fold (resulting K_d_ in the picomolar range), without increasing cross-reactivity [85]. In vitro, this increases the binding half-life of ImmTAC molecules to specific antigens, from seconds to hours [79,85,86].

Thirdly, binding of the ImmTAC molecule to a cancer-associated antigen needs to be coupled to immune cell activation. This is achieved by creating a TCR-anti-CD3 scFv domain fusion protein, which engages the CD3 receptors on T cells, replicating a natural immune synapse. The anti-CD3 domain of an ImmTAC molecule has a nanomolar affinity for the CD3 receptor that is a 1000-fold weaker than the TCR domain, ensuring that the affinity for the pHLA complex drives ImmTAC molecule activity, rather than the ability to stimulate T cells through the CD3 receptor [79].

**Figure 2:**
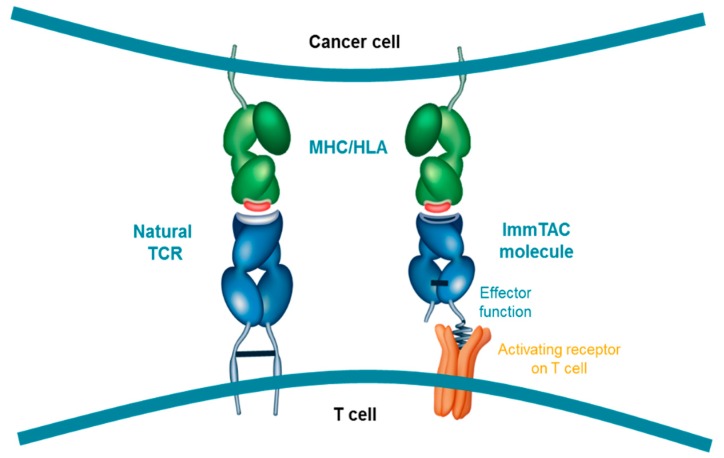
ImmTAC molecules are designed to mimic the natural immune synapse formed by interaction of a TCR with a peptide-human-leukocyte-antigen (pHLA) complex. The anti-CD3 effector function attracts and binds to CD3 receptors (activating receptor) on T cell surfaces triggering T-cell mediated cancer cell lysis; HLA, Human leukocyte antigen; MHC, Major histocompatibility complex; TCR, T cell receptor.

The resulting ImmTAC molecule is a soluble, high-affinity TCR that targets a specific pHLA complex. Importantly, unlike adoptive T cell therapies, ImmTAC molecules are not patient-specific and as a result can be administered ‘off-the-shelf’, reducing the delay to patients and the need for specialised cellular manufacturing. However, they are currently only effective in patients expressing the specific HLA subtype that each ImmTAC molecule is designed to target, restricting their use to those patients. By binding a specific pHLA complex on the surface of tumour cells, the ImmTAC molecule can recruit CD3+ T cells (regardless of their native TCR-directed specificity), leading to lysis of the target cell. The intention behind the design of the ImmTAC platform was to mimic the immunological synapse that forms from native TCRs binding to pHLA complexes (Figure 2). Cytokine release by the T cell further stimulates the local immune response [79].

## 6. MoA of Tebentafusp

Tebentafusp is an ImmTAC molecule that targets a fragment of the melanocytes lineage-specific antigen gp100_280–288_ (alternative names Melanocyte protein Pmel17, melanoma-associated ME20 antigen, ME20-M, [UniProtKB-P40967]) presented by HLA-A*02:01 [87]. The target, gp100, was first identified as melanoma associated by the isolation of melanoma-specific cytotoxic T lymphocytes that recognised gp100 fragments presented by HLA [88]. Subsequent RNA expression analysis revealed that gp100 is expressed strongly in melanomas, weakly in normal melanocytes and minimally in non-melanocyte cells [89,90]. The gp100 fragment targeted by tebentafusp, gp100_280–288_, has particular affinity for the HLA-A subtype HLA-A*02:01 [91]. The HLA-A*02:01–gp100_280–288_ peptide complex is, therefore, an attractive target for anti-melanoma therapy [89,90]; however, a limitation is that only about 50% of Caucasian individuals are HLA-A*02:01 positive [92]. Based on its MoA, tebentafusp is not effective in HLA-A*02:01-negative patients.

**Figure 3:**
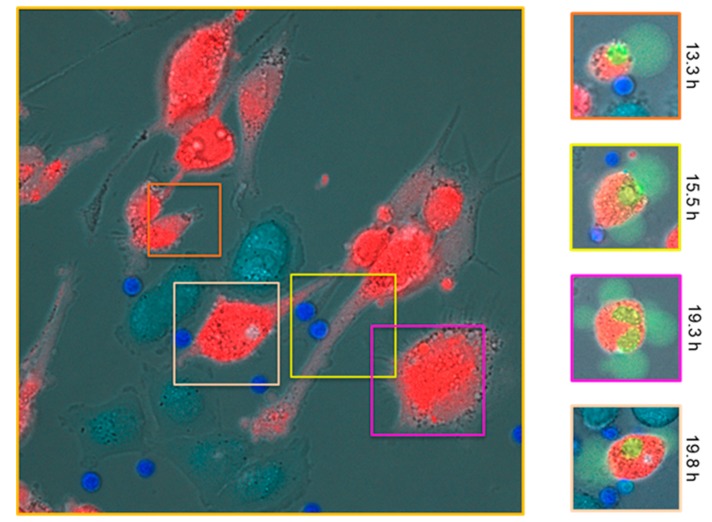
Images from time-lapse video microscopy taken in dose-response experiments in vitro conducted over a range of successive time points from 0–20 h showing tebentafusp (80 pM)-mediated killing of gp100-positive HLA-A*02:01-positive uveal melanoma (UM) cells (92-1; red) by CD8+ T cells (dark blue). Melanoma cells that are gp100-negative, HLA-A*02:01-positive—an HLA-matched control—(A375; pale blue) are ignored by CD8+ T cells. The main image (left) represents *t* = 0 in the assay and is captured with a 63× oil immersion objective on Zeiss Axiovert 200M inverted microscope. The areas of green shown in the time-lapse images on the right-hand side are a dye activated by active caspase 3/7 to bind DNA and fluoresce, highlighting cellular apoptosis as a direct result of on-target tebentafusp-mediated T-cell redirection. The times where apoptosis of the highlighted target cells are first observed are denoted on the right and the border colours correspond to the same colour square in the main (left) image. Note the change in morphology of the cells on the right in line with the observed apoptosis as indicated by the green dye; UM, uveal melanoma.

Preclinical data with tebentafusp supported a MoA based on T cell-mediated killing of gp100+/HLA-A*02:01+ cell lines [93]. These data showed that when incubated with gp100+ cancer cell lines and tebentafusp, CD8+ T cells show lytic activity and cytokine production that are not seen in the absence of tebentafusp [94]. Furthermore, lytic activity is restricted to only gp100-positive and HLA-A*02:01-positive cancer cell lines (Figure 3) [94]. Further in vitro studies showed that antitumour activity is not restricted to CD8+ T cells, and that CD4+ T cells are also directed to lyse gp100+ cancer cell lines in the presence of tebentafusp [87]. Additionally, T cells engaged by tebentafusp secrete high levels of tumour necrosis factor α (TNFα), IL-2, IL-6 and interferon γ (IFNγ) [87]. TNFα and IFNγ are strong pro-inflammatory agents that further promote cancer cell apoptosis, attracting and activating lymphocytes and inducing maturation of dendritic cells in the process [87]. Furthermore, tebentafusp has been shown by in vitro studies to potentiate ‘epitope spreading’ whereby tumour-associated antigens released by apoptotic tumour cells are captured and displayed by dendritic cells, which then engage T cells to lyse more cancer cells [95].

Emerging from the preclinical data is a picture of the MoA of tebentafusp. On a molecular level, tebentafusp induces formation of an immune synapse between a T cell and a tumour cell to cause tumour lysis. T cells are activated in a polyclonal manner, regardless of the specificity of their native TCR. On a broader level, this process might be self-sustaining as the engaged T cells produce a range of pro-inflammatory cytokines, and the surrounding dendritic cells take up tumour-associated antigens from killed cells and present these to lymphocytes. Both these processes might further serve to amplify the immune response to tumours [87].

## 7. Tebentafusp in Clinical Studies

**Figure 4:**
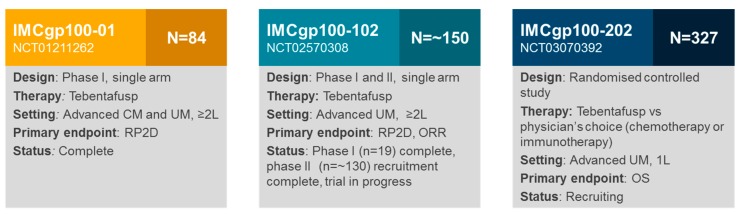
Clinical studies with tebentafusp in UM; 1L, First line; 2L, Second line; CM, Cutaneous melanoma; ORR, Objective response rate; RP2D, Recommended phase II dose; UM, Uveal melanoma.

To date, two clinical studies with tebentafusp in UM have reported results (IMCgp100-01 and IMCgp100-102 phase I) [4,5], and there are still two ongoing trials (IMCgp100-102 phase II, IMCgp100-202; Figure 4).

The IMCgp100-01 study was the first-in-human study of tebentafusp in patients with advanced or metastatic melanoma, who had received any number of prior therapies (*N* = 84; a cohort with UM was included, *n* = 16) [5]. The primary objective was to determine a recommended phase II dose for tebentafusp; safety and efficacy of the therapy were secondary objectives. Two partial responses in patients with mUM (14%; *n* = 14 evaluable) were observed, and eight patients (57%) with mUM achieved disease control for ≥ 16 weeks (5). In the metastatic CM cohort (*n* = 33 evaluable), two partial responses (6%) and six instances of a stable disease (18%) were observed [5]. These results were the first indication that tebentafusp had clinical activity in melanoma. Furthermore, this clinical activity was observed in mUM, which, as mentioned, is usually resistant to other forms of therapy. Based on this activity in mUM, IMCgp100-102 was initiated to study patients with mUM exclusively. To date, results are available for the phase I arm of this trial—3/17 (18%) patients achieved a partial response and 11/17 (65%) achieved disease control for ≥ 16 weeks [4]. These results underline the promising efficacy seen in the previous trial, and justify the investigation of tebentafusp as first-line therapy for mUM in the pivotal IMCgp100-202 trial.

The safety profile of tebentafusp was consistent across both the IMCgp100-01 and IMCgp100-102 (phase I arm) studies. In both of these studies, the most frequent treatment-related adverse events (AEs) were in skin or were likely cytokine-mediated. Common events included rash, pruritus, dry skin, pyrexia, hypotension, periorbital oedema, fatigue, nausea and chills [4,5]. It is likely that these AEs represent on-target activity of tebentafusp, comprising skin-related AEs from targeting of gp100-positive melanocytes and cytokine-related AEs from tebentafusp-mediated cytokine release. Investigators report that these AEs are manageable with standard clinical interventions—intravenous fluids and corticosteroids have been used to treat hypotension; paracetamol and anti-histamines have been used to alleviate skin toxicities and pyrexia. Cytokine release syndrome (CRS) has been observed in tebentafusp clinical trials, with Grade ≥ 3 CRS reported by investigators in < 5% of patients. CRS was reversible through medical management, including corticosteroid therapy and select measures to address patient symptoms; no patient deaths from CRS have been observed [4,5].

Based on investigator experience, treatment-related AEs with tebentafusp typically develop within 2–12 h, following the end of infusion, and generally abate within 48–72 h after onset, with or without treatment. They are also more common with the first three doses, with AEs appearing to be less frequent and less severe from the fourth dose onwards [96]. To minimise this toxicity, an intra-patient dose escalation regimen was introduced, based on the IMCgp100-01 trial—patients receive 20 µg tebentafusp on day 1, 30 µg on day 8 and 68 µg weekly, thereafter [4]. For the first three doses, patients are kept in hospital overnight for monitoring and treatment of any AEs.

**Figure 5:**
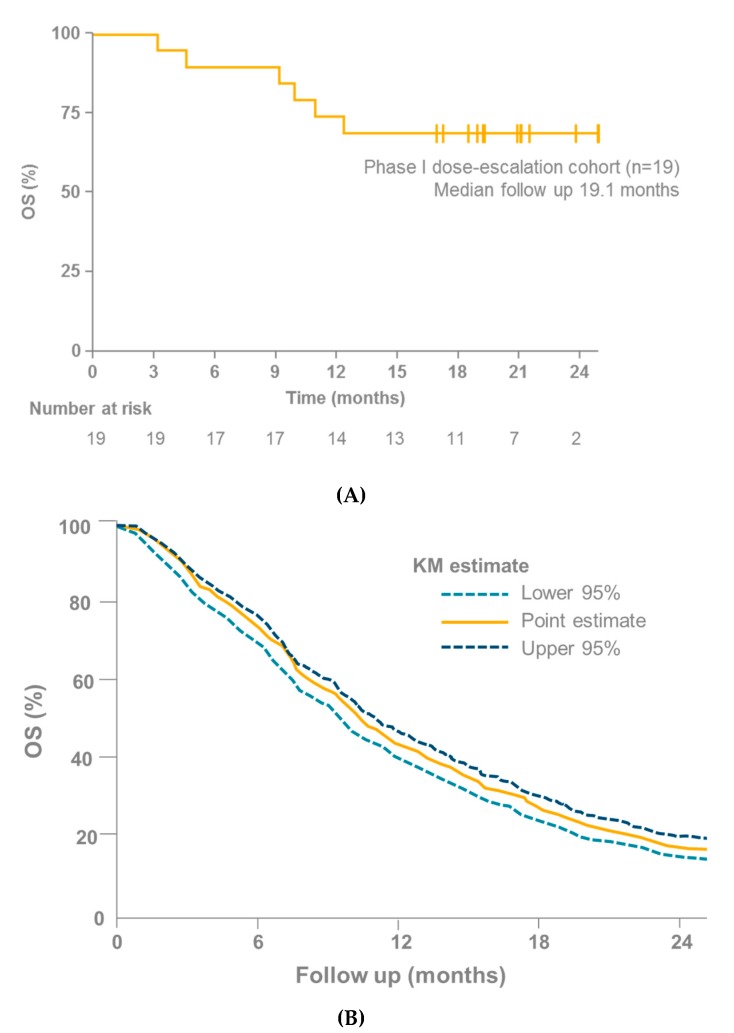
(**A**) OS in 19 patients with mUM receiving tebentafusp as part of the IMCgp100-102 trial [4]. (**B**) OS in 915 patients with mUM receiving immunotherapy, kinase inhibitors, anti-angiogenic therapy, chemotherapy or liver-directed therapy from 29 different trials [42]; KM, Kaplan–Meier; mUM, Metastatic uveal melanoma; OS, Overall survival.

In light of the lack of efficacy shown by checkpoint inhibitors in mUM, the reported ORR of 14–18% and median PFS of 3.7–5.6 months with tebentafusp in mUM are encouraging (although the number of patients who have received tebentafusp and those for whom data are available is small; *n* = 34) [4,5,95]. Particularly encouraging is the overall survival (OS) data from the IMCgp100-01 and IMCgp100-102 trials, especially when compared with other studies in mUM (OS was a secondary endpoint in IMCgp100-01 and IMCgp100-102). Three meta-analyses have recently been published that investigate cohorts of patients with mUM (Table 2) [3,41,42]. These analyses considered a number of studies, covering a range of therapies in patients with mUM. None of these studies reported an OS rate at 1 year > 55% [3,41,42]. In contrast, the OS rates at 1 year in the IMCgp100-01 and IMCgp100-102 (phase I arm) studies were 73% (95% confidence interval [CI] 38–91) [95] and 74% (95% CI 48–88) [4], respectively (Figure 5).

Interestingly, patients who experienced Grade ≥ 2 skin toxicities within 21 days of their first tebentafusp infusion, trended towards longer survival than patients who did not develop these AEs, and patients in the top three quartiles for reduction in arterial pressure trended towards longer survival than those in the lower quartile [4]. It is worth noting that these analyses were conducted in a small number of patients (*n* = 42) and need confirming in larger studies. However, the association is nevertheless interesting, and suggests that some of the more common AEs observed with tebentafusp reflect the ability of the patient’s immune system to mount effective immune responses.

It is interesting to speculate on why tebentafusp shows promising activity in otherwise hard-to-treat mUM. There are two potential explanations—first, gp100 expression is particularly high in UM [97,98]. Preclinical studies with tebentafusp showed that target cells displaying a higher surface concentration [or density] of gp100 peptide-HLA complexes result in a greater degree of target cell lysis [93]. On the basis of this finding, it is reasonable to hypothesise that greater gp100 expression translates to a more efficient cancer cell killing in vivo. Second, UM appears to have a relatively non-T cell-inflamed genetic signature [74]. Non-T cell-inflamed tumours show a lower expression of genes associated with T cell infiltration and localised inflammation, compared with T cell-inflamed tumours [99]. This might be related to the lower mutational burden and low antigenicity of UM [73,74]. Lack of T cell inflammation might be a barrier to immune-mediated cancer destruction; therefore by recruiting T cells to antigen-positive sites and inducing an inflammatory response tebentafusp might overcome this barrier.

## 8. Future Prospects

Several studies with tebentafusp in both mUM and metastatic CM are ongoing. Recruitment for the dose expansion phase of IMCgp100-102 (a Phase II, single arm open-label study in previously treated patients with mUM) is complete, and the trial will further investigate the efficacy and safety of tebentafusp. IMCgp100-202 is a pivotal, randomised, controlled, open-label study of tebentafusp versus the physician’s choice of therapy (dacarbazine, ipilimumab [anti-CTLA-4] or pembrolizumab [anti-PD-1]) in previously untreated patients with mUM. This study is currently recruiting patients in a number of locations across North America, Europe and Australia. A study in metastatic CM of tebentafusp ± durvalumab (anti-PD-L1) ± tremelimumab (anti-CTLA-4) is also ongoing (IMCgp100-201; ClinicalTrials.gov Identifier: NCT02535078).

There would seem to be a scope for investigating tebentafusp as an adjuvant therapy for patients whose genetic tumour profile indicates a high risk of developing metastatic disease. There might also be a scope for investigating tebentafusp as a neoadjuvant therapy for primary UM, to reduce the size of the tumour before radiotherapy or resection, hence minimising collateral damage to healthy ocular tissues.

Beyond tebentafusp, a clear next step would be to produce additional anti-gp100 ImmTAC molecules that target other HLA subtypes, exploring the benefit of tebentafusp in patients who are HLA-A*02:01-negative. Furthermore, the encouraging data seen with tebentafusp suggest that an ImmTAC platform-based approach could bring benefit in other, non-UM settings, to patients who do not currently respond to immunotherapy. Key to bringing a benefit to other settings will be the identification of suitable peptide antigens for new ImmTAC molecules to target.

## 9. Conclusions

ImmTAC molecules are a first-in-kind therapeutic agent that re-directs T cells against cancer cells, avoiding the limitations of adoptive T cell therapy. Tebentafusp shows promising clinical activity in patients with mUM, with survival rates that appear superior to those reported with other treatments.

## Figures and Tables

**Table 1:** Existing therapies for metastatic uveal melanoma (mUM).

Therapy Class	Therapy Examples	Efficacy in mUM
Liver-directed therapies	Surgical resection	Surgical resection of isolated liver deposits appears to result in more favourable survival than no resection [31].
	Bland embolisation Chemoembolisation Radioembolisation Immunoembolisation	Limited prospective data for non-surgical therapies. Response rates have been superior to systemic chemotherapy [32,33], but only liver sites are targeted. Results with liver-directed melphalan have been particularly impressive in patients with liver metastases from mUM (ORR 47%) [34].
Chemotherapy	Dacarbazine Temozolomide Cisplatin Treosulfan Fotemustine Various combinations	Results to date have been disappointing [35].
Kinase inhibitors	Sorafenib (multikinase inhibitor)	A randomised phase II trial of sorafenib showed PFS to be superior to placebo (median 5.5 vs 1.9 months) [36].
	Selmutinib (MEK inhibitor)	A randomised phase III trial of selmutinib + dacarbazine failed to show a benefit, compared to placebo + dacarbazine (ORR 3% vs 0%, respectively), in contrast to promising phase II results [37,38].
	Trametinib (MEK inhibitor)	A phase I trial of trametinib demonstrated limited clinical activity (0% ORR, 50% achieved stable disease) [39].
	Sunitinib (multikinase inhibitor)	A phase II trial of sunitinib showed no benefit vs dacarbazine (ORR 0% vs 8%, respectively) [40].
Immunotherapy	Pembrolizumab (PD-1 inhibitor) Nivolumab (PD-1 inhibitor) Ipilimumab (CTLA-4 inhibitor)	A meta-analysis of mUM trials and a real-world study both concluded limited benefit of checkpoint inhibitors in mUM (median PFS 2.6–2.8 months) [41,42].

CTLA-4—Cytotoxic T-lymphocyte antigen 4; mUM—Metastatic uveal melanoma; ORR—overall response rate; PD-1—Programmed cell death protein-1; PFS—Progression-free survival.

**Table 2:** Overall survival (OS) data with tebentafusp compared with results from three meta-analyses looking at a range of therapies for mUM.

Study	N Total	Number of Studies	Therapy	Median OS (months)	OS Rate at 1 Year (%)
Algazi, 2016 [41]	56	9	Anti-PD-1 or Anti-PD-L1 antibodies	7.7	~45
Khoja, 2016 [42]	915	29	Immunotherapy, kinase inhibitors, anti-angiogenic agent, intra-hepatic chemotherapy or immunotherapy, LDTs	10.2	43
Rantala, 2019 [3]	2494	78	Immunotherapy, chemotherapy, LDTs, surgery	12.8	52
IMCgp100-01 [96]	15 (evaluable in UM cohort)	1	Tebentafusp	Not reached after 16 months follow-up	73
IMCgp100-102 [4]	19	1	Tebentafusp	Not reached after 16 months follow-up	74

LDT, Liver-directed therapy; mUM, Metastatic uveal melanoma; OS, Overall survival; PD-1, Programmed cell death protein-1; PD-L1, Programmed death-ligand 1; UM, Uveal melanoma.
